# EGFR and COX-2 Dual Inhibitor: The Design, Synthesis, and Biological Evaluation of Novel Chalcones

**DOI:** 10.3390/molecules27041158

**Published:** 2022-02-09

**Authors:** Arafa Musa, Ehab M. Mostafa, Syed Nasir Abbas Bukhari, Nasser Hadal Alotaibi, Ahmed H. El-Ghorab, Amr Farouk, AbdElAziz A. Nayl, Mohammed M. Ghoneim, Mohamed A. Abdelgawad

**Affiliations:** 1 Department of Pharmacognosy, College of Pharmacy, Jouf University, Sakaka 72341, Saudi Arabia; akmusa@ju.edu.sa (A.M.); emmoustafa@ju.edu.sa (E.M.M.); 2 Department of Pharmaceutical Chemistry, College of Pharmacy, Jouf University, Sakaka 72341, Saudi Arabia; sbukhari@ju.edu.sa; 3 Department of Clinical Pharmacy, College of Pharmacy, Jouf University, Sakaka 72341, Saudi Arabia; nhalotaibi@ju.edu.sa; 4 Department of Chemistry, College of Science, Jouf University, Sakaka 72341, Saudi Arabia; aghorab@ju.edu.sa (A.H.E.-G.); aanayel@ju.edu.sa (A.A.N.); 5 Flavour and Aroma Chemistry Department, National Research Centre, Giza 12622, Egypt; amrfarouk01@gmail.com; 6 Department of Pharmacy Practice, College of Pharmacy, AlMaarefa University, Ad Diriya 13713, Saudi Arabia; mghoneim@mcst.edu.sa

**Keywords:** EGFR, COX-2, kinase, anticancer, anti-inflammatory

## Abstract

For most researchers, discovering new anticancer drugs to avoid the adverse effects of current ones, to improve therapeutic benefits and to reduce resistance is essential. Because the COX-2 enzyme plays an important role in various types of cancer leading to malignancy enhancement, inhibition of apoptosis, and tumor-cell metastasis, an indispensable objective is to design new scaffolds or drugs that possess combined action or dual effect, such as kinase and COX-2 inhibition. The start compounds A1 to A6 were prepared through the diazo coupling of 3-aminoacetophenone with a corresponding phenol and then condensed with two new chalcone series, C7–18. The newly synthesized compounds were assessed against both COX-2 and epidermal growth factor receptor (EGFR) for their inhibitory effect. All novel compounds were screened for cytotoxicity against five cancer cell lines. Compounds C9 and G10 exhibited potent EGFR inhibition with IC_50_ values of 0.8 and 1.1 µM, respectively. Additionally, they also displayed great COX-2 inhibition with IC_50_ values of 1.27 and 1.88 µM, respectively. Furthermore, the target compounds were assessed for their cytotoxicity against pancreatic ductal cancer (Panc-1), lung cancer (H-460), human colon cancer (HT-29), human malignant melanoma (A375) and pancreatic cancer (PaCa-2) cell lines. Interestingly, compounds C10 and G12 exhibited the strongest cytotoxic effect against PaCa-2 with average IC_50_ values of 0.9 and 0.8 µM, respectively. To understand the possible binding modes of the compounds under investigation with the receptor cites of EGFR and COX-2, a virtual docking study was conducted.

## 1. Introduction

**Figure 1:**
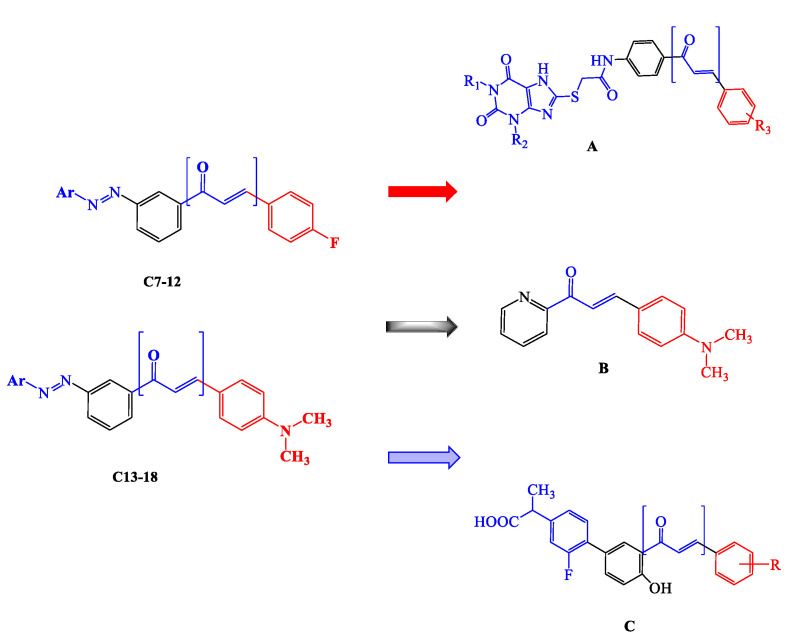
Structure of reported compounds (A, B and C) and target compounds (C7–18).

**Scheme 1:**
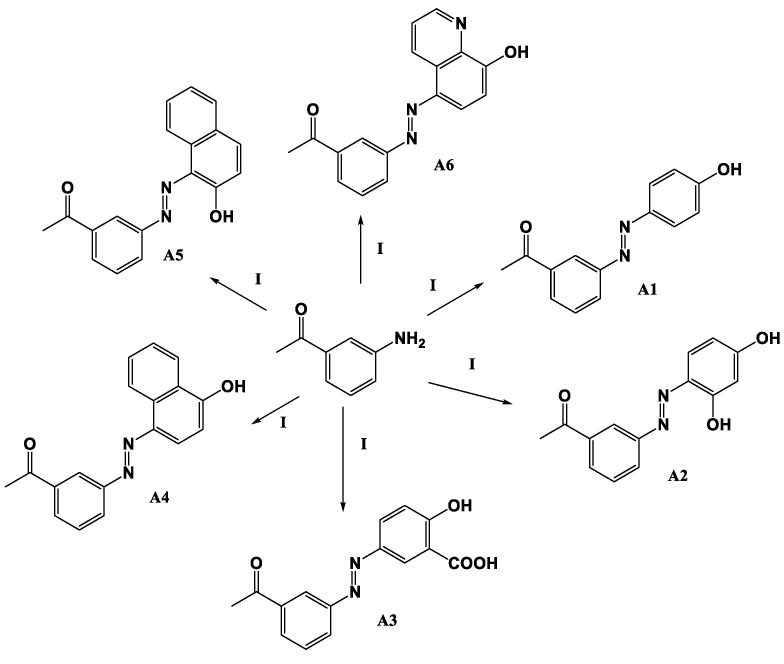
The synthetic pathway for A1 to A6.

**Scheme 2:**
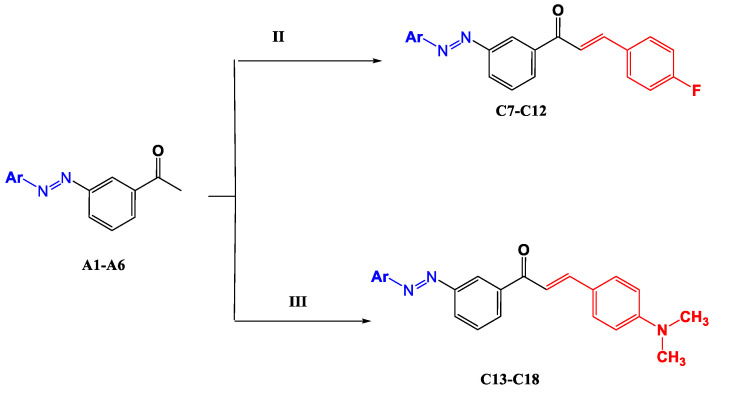
The synthetic pathway for C7–C18.

**Scheme 3:**
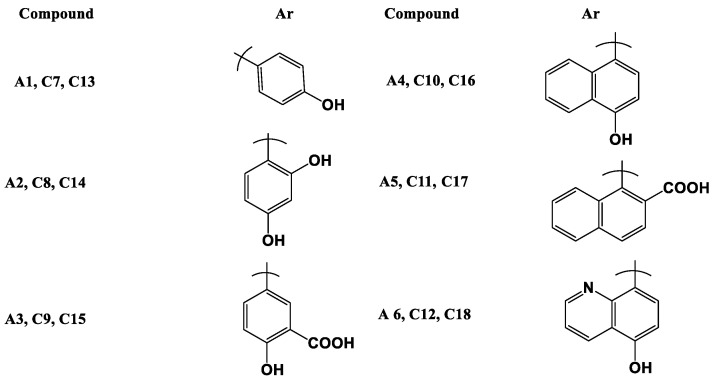
The structures of substituents in compounds A1–A6 and C7–C18.

Cancer is the second-highest cause of death in the United States and a serious public health concern globally. The coronavirus disease 2019 (COVID-19) pandemic had a negative impact on cancer diagnosis and therapy in 2020 because health care facilities were shut down out of fear of exposure, which may have caused a short-term decrease in cancer incidence but also may have caused an increase in advanced-stage disease and death [1]. Radiation therapy, surgery, and chemotherapy are all used in combination or separately to treat cancer, yet it may go undetected and untreated for many years [2]. The urgent need for novel anticancer treatments that are less expensive and have fewer side effects is obvious [3]. Numerous therapies have been approved, including selective cyclooxygenase-2 (COX-2) inhibitors and epidermal growth factor receptor (EGFR) monoclonal antibodies. Several studies have revealed that EGFR and COX-2 signaling pathways work together to promote cancer. This has been cited in support of a combinatorial strategy that specifically targets these two routes. Although COX-2 inhibitors have been shown to have a stronger antitumor impact when coupled with other agents, they potentially have deadly cardiac consequences [4,5]. Overexpression of cyclooxygenase-2 (COX-2) has been linked to the initiation and progression of a variety of common major malignancies, including lung, breast and colon cancer [6,7,8]. Tumor COX-2 expression may play an important role in tumor resistance to apoptosis, angiogenesis, invasion and immune suppression [9]. On the other hand, EGFR is a tyrosine kinase (TK) receptor that is activated when it is bound to the Epidermal Growth Factor and other growth factor ligands. This activates a number of downstream pathways that control cell processes such as DNA synthesis and proliferation [10,11]. Therefore, it has become a major target for various cancer therapies. First, EGFR inhibitors through the third generation have shown strong effectiveness and given hope to patients, but all of them developed resistance during the first 9–15 months following therapy, and no significant advances in resistance to third-generation inhibitors has been made [12]. New and more effective chemotherapeutics that target multiple cancer-related pathways at the same time by using multi-target drugs could be developed [13]. Therefore, development of such drugs to act as both kinase and COX inhibitors is predicted to be very effective and selective in treating cancer [14,15,16,17,18,19]. Among the highly effective and reactive organic scaffolds is the chalcone nucleus, which has anticancer, antioxidant, anti-inflammatory, and analgesic effects [20,21,22,23]. It can be used to synthesize and develop certain lead compounds that have reasonable plasma concentrations and low toxicity. The diazineyl linkage has dual action; its pharmacokinetic effect enhances absorption, and its pharmacodynamic may lead to a reduction in NH–NH by liver enzymes causing increased activity and decreased toxicity to facilitate its excretion [16]. Therefore, the aim of this study is to design a new scaffold containing chalcone with promising anticancer effect through the inhibition of kinase and COX enzymes (Scheme 1, Scheme 2 and Scheme 3 and Figure 1).

The target compounds were prepared according to the following schemes.

Reagent and conditions: (I) (a) 3-aminoacetophenone, HCl, NaNO_2_, stirring at 0 °C for 4 h; (b) aromatic phenol, KOH, stirring for 24 h.

Reagent and conditions: (II) A1 to A6, p-fluorobenzaldehyde, KOH, stirring at room temperature for 72 h; (III) A1 to A6, p-dimethylaminebenzaldehyde, NaOH, stirring at room temperature for 72 h;

## 2. Results and Discussion

### 2.1. Chemistry

(E)-1-(3-((2,4-Dihydroxyphenyl)daizenyl)phenyl)ethan-1-one (A2)

Red crystals, mp (280–282 °C), ^1^H NMR at ẟ; 11.83 (s, H, OH), 8.50 (s, H, OH) 8.32–8.01 (m, 4H, acetophenone), 7.99–7.53 (m, 3H, resorcinol), 2.52 (s, 3H, CH_3_), ^13^C NMR; 185.15, 145.12, 144.23, 138.45, 137.25, 134.52, 131.24, 128.29, 125.23, 120.14, 117.29, 108.65, 107.44, 31.62. IR (cm^−1^), 1606 (C=C), 1708 (C=O), 3110 (C=CH), 3520 (OH). Element Analysis Calculated of C_14_H_12_N_2_O_3_, C; 65.62, H; 4.72, N; 10.93: Present C; 65.50, H; 4.80, N; 11; EIMS: *m*/*z* (%): 256 (5.45%) [M]+.

(E)-5-((3-Acetylphenyl)diazenyl)-2-hydroxybenzoic acid (A3)

Dark red crystals, mp (311–313 °C), ^1^H NMR at ẟ; 14.25 (s, 1H, COOH), 11.24 (s,1H, OH), 8.39 (s,1H, ArH), 8.13–8.11 (m, 2H, ArH), 7.77–7.73 (m. 2H ArH), 7.18–7.16 (m, 2H, ArH), 2.51 (s, 3H, CH_3_); ^13^C NMR; 198.08, 171.70, 152.42, 144.69, 138.40, 130.78, 130.44, 129.46, 128.44, 127.18, 126.91, 126.52, 122.33, 118.94, 27.47; IR (cm^−1^), 1680 (C=O). 3600–2600 (COOH). Element Analysis Calculated of C_15_ H_12_ N_2_O_4_, C; 63.38, H; 4.25, N; 9.85: present C; 63.40, H; 4.30, N; 9.80. EIMS: *m*/*z* (%): 284 (23.20%) [M]+.

(E)-1-(3-((4-Hydroxynaphthalen-1-yl)diazenyl)phenyl)ethan-1-one (A4)

Dark brown crystals, mp (322–324 °C), ^1^H NMR, 8.74 (s, 1H, OH), 8.40–8 (m, 2H, ArH), 7.92–7.88 (m, 1H, ArH), 7.68–7.52 (m, 1H, ArH), 7.52–7.42 (m, 2H, ArH), 7.40–7.14 (m, 4H, ArH), 2.51 (s, 3H, CH_3_). ^13^C NMR; 198.76, 178.91, 155.64, 138.11, 131.08, 129.56, 129.52, 127.96, 125.40, 124.66, 124.34, 122.04, 121.85, 120.53, 119.44, 117.64, 114.91, 27.42. IR, (cm^−1^), 1600 (C=C), 1700 (C=O), 3400 (OH); Element Analysis Calculated of C_18_ H_14_ N_2_O_2_, C; 74.47, H; 4.86, N; 9.65; Present C; 74.40, H; 4.90, N; 9.70; EIMS: *m*/*z* (%): 290 (17.83%) [M]+.

(E)-1-(3-((2-Hydroxynaphthalen-1-yl)diazenyl)phenyl)ethan-1-one (A5)

Brownish-red crystals, mp (313–315 °C), ^1^H NMR, at ẟ 15.60 (s, IH, OH), 8.55 (d, *J* = 8 Hz, 1H, ArH), 8.35 (s, 1H, ArH), 8.15 (d, *J* = 7.6 Hz, 1H, ArH), 7.99–7.92(m, 1H, ArH), 7.80 (d, *J* = 7.6, 1H, ArH) 7.71–7.63 (m, 2H, ArH), 7.50–7.48 (m, 2H, ArH), 6.92 (d, *J* = 9.6 Hz, 1H, ArH), 2.51 (s, 3H, CH_3_); ^13^C NMR, 198.03, 170.78, 145.95, 141.03, 138.62, 133.21, 130.69, 130, 129.74, 129.47, 128.35, 127.47, 126.53, 124.86, 123.08, 121.90, 118.97, 27.47; IR; 1690 (C=O), 2950 (CH), 3100 (C=CH), 3450 (OH); Element Analysis Calculated of C_18_H_14_N_2_O_2_, C, 74.47; H, 4.86; N, 9.65; Present C, 74.50; H, 4.90; N, 9.60. EIMS: *m*/*z* (%): 290 (23.36%) [M]+.

(E)-1-(3-((8-Hydroxyquinolin-5-yl)diazenyl)phenyl)ethan-1-one (A6)

Reddish-brown crystals, mp (293–295 °C), ^1^H NMR at ẟ; 9.35 (s, IH, OH), 8.35–8.29 (m, 1H, ArH), 8.20–8.09 (m, 2H, ArH), 7.96–7.96 (m, 2H, ArH), 7.67–7.57 (m, 2H, ArH), 6.80–6.72 (m, 2H, ArH), 2.51 (s, 3H, CH_3_), ^13^C NMR; 198.41, 188.72, 154.02, 141.24, 138.34, 136.48, 135.28, 130.08, 129.44, 127.65, 126.39, 125.81, 120.27, 119.24, 114.20, 110.76, 27.48; IR; 1580 (C=C), 1620 (C=C), 1710 (C=O), 3350 (OH); Element analysis calculated of C_17_H_13_N_3_O_2_, C, 70.09; H, 4.50; N, 14.42; Present C, 70.10; H, 4.60; N, 14.60. EIMS: *m*/*z* (%): 291 (47.96%) [M]+.

(E)-3-(4-Fluorophenyl)-1-(3-((E)-(4-hydroxyphenyl) diazenyl)phenyl) prop-2-en-1-one (C7)

Brown crystals, mp (decomposition 317 °C), ^1^H NMR at ẟ 10.45 (s, 1H, OH), 8.52 (s, 1H, ArH), 8.30 (d, *J* = 7.6 Hz, 1H, ArH), 8.09–8.01 (m, 4H, ArH), 7.90–7.75 (m, 4H, ArH), 7.35–7.31 (m, 3H, ArH), 7.12 (d, *J* = 12 Hz, 1H, ArCH); ^13^C NMR; 188.17, 165.24, 162.77, 161.87, 152.87, 145.66, 143.87, 139.15, 132.02, 131.93, 131.80, 130.72, 130.42, 130, 126.27, 125.66, 122.62, 122.30, 116.56, 116.50, 116.35; IR; 1570 (C=C), 1610 (C=C), 1680 (C=O), 3370 (OH); Element analysis calculated of C_21_H_15_FN_2_O_2_, C, 72.82; H, 4.37; N, 8.09; Present C, 72.90; H, 4.40; N, 8.10. EIMS: *m/z* (%): 346 (42.06%) [M]+.

(E)-1-(3-((E)-(2,4-Dihydroxyphenyl) diazenyl) phenyl)-3-(4-fluorophenyl) prop-2-en-1-one (C8)

Brown crystals, mp (decomposition 323 °C), ^1^H NMR, at ẟ 12.30, 8.58 (s, 2H, 2OH), 8.52 (s, IH, ArH), 8.25–8.02 (m, 5H, ArH), 8–7.95 (m, 3H, ArH), 7.85–7.73 (m, 2H,), 6.52–6.51 (*d, d*, J_1_ = 9.2, J_2_ = 8.8 Hz, 2H, ArH); ^13^C NMR; 187.18, 164.26, 163.14, 161.64, 151.84, 151.64, 149.99, 145.66, 143.84, 141.74, 132.97, 131.90, 129.60, 127.26, 126.44, 125.82, 122.54, 120.37, 119.88, 116.58, 116.36, 109.99, 103.46; IR (cm^−1^), 1620 (C=C), 1680 (C=O), 3060 (C=CH), 3440 (OH); Element analysis calculated of C_21_H_15_N_2_O_3_, C, 69.61; H, 4.17; N, 7.73; Present C, 69.60; H, 4.20; N, 7.90. EIMS: *m/z* (%): 362 (24.37%) [M]+.

5. -((Z)-(3-((E)-3-(4-Fluorophenyl)acryloyl)phenyl)diazenyl)-2-hydroxybenzoic acid (C9)

Red crystals, mp (decomposition 327 °C), ^1^H NMR, at ẟ 13.65(s, 1H, COOH), 12.3 (s, 1H, OH), 8.57 (s, 1H, ArH), 8.42–8.28(m, 3H, ArH), 8.10–8.01(m, 4H, ArH), 7.38–7.30 (m, 3H, ArH), 7.10–7.04 (m, 2H, ArH); ^13^C NMR; 185.48, 165.26, 153.44, 151.42, 146.12, 144.20, 141.21, 140.24, 139.61, 138.22, 136.41. 133.61, 131.12, 130.51, 129.42, 128.52, 127.28, 126.32, 125.41, 122.10, 117.22, 116.92, 116.31; IR; 2500–3500 (COOH), 1600 (C=C); 1690 (C=O); Element Analysis Calculated of C_22_H_15_FN_2_O_4_, C, 67.69; H, 3.87; N, 7.18; Present C, 67.60; H, 3.90; N, 7.20. EIMS: *m*/*z* (%): 390 (15.25%) [M]+.

(E)-3-(4-Fluorophenyl)-1-(4-((E)-(4-hydroxynaphthalen-1-yl)diazenyl)phenyl) prop-2-en-1-one (C10)

Dark red crystals, mp (319–321 °C), ^1^H NMR at ẟ 11.30 (s, 1H, OH), 8.25–8.05 (m, 4H, ArH), 7.98–7.93 (m, 3H, ArH), 7.87–7.81 (m, 4H, ArH), 7.78–7.60 (m, 3H, ArH), 7.37–6.78 (m, 2H, ArH); ^13^C NMR; 187.45, 154.26, 152.48, 149.45, 148.57, 147.25, 142.24, 140.34, 139.47, 138.65, 137.45, 138.14, 136.45, 132.68, 131.28, 129.48, 128.46, 125.35, 124.46, 122.13, 120.34, 119.48, 118.24, 116.34, 115.45; IR (cm^−1^) 1700 (C=O), 3060 (C=CH), 3350(OH); Element analysis, calculated of C_25_H_17_FN_2_O_2_, C, 75.75; H, 4.32; N, 7.07; Present C, 75.70; H, 4.40; N, 7.10. EIMS: *m*/*z* (%): 396 (1.47%) [M]+.

(E)-3-(4-Fluorophenyl)-1-(4-((E)-(2-hydroxynaphthalen-1-yl)diazenyl)phenyl)prop-2-en-1-one (C11)

Dark red crystals, mp (312–3141 °C), ^1^H NMR 15.72 (s,1H, OH), 8.54–8.51 (m, 2H, ArH), 8.20–8.13 (m, 2H, ArH), 8.03–7.98 (m, 2H, ArH), 7.85–7.75 (m, 3H, ArH), 7.66–7.64 (m, 2H, ArH), 7.50–7.48 (m, 2H, ArH), 7.34–7.32(m, 2H, ArH), 6.93(d, J = 9.2, 1H); ^13^C NMR; 188.99, 177.30, 171.06, 165.43, 162.81, 145.80, 143.86, 141.25, 139.39, 133.19, 131.96, 131.88, 130.86, 129.99, 129.80, 129.52, 128.44, 128.44, 127.90, 126.70, 124.79, 122.54, 121.95, 119.70, 116.37; IR (cm^−1^) 1520 (C=C), 1685 (C=O), 3400 (OH); Element Analysis Calculated of C_25_H_17_FN_2_O_2_, C, 75.75; H, 4.32; N, 7.07; Present C, 75.80; H, 4.30; N, 7.00; EIMS: *m*/*z* (%): 396 (76.94) [M]^+^.

(E)-3-(4-Fluorophenyl)-1-(4-((E)-(8-hydroxyquinolin-5-yl)diazenyl)phenyl)prop-2-en-1-one (C12)

Dark reddish-brown crystals, mp (316–318 °C), ^1^H NMR at ẟ 9.36 (d, *J* = 8.4 Hz, 1H, ArH), 9.02 (s, 1H, OH), 8.32–8.20 (m, 2H, ArH), 8.08–8.05 (m, 3H, ArH), 7.87–7.80 (m, 2H, ArH), 7.37–7.27 (m, 4H, ArH), 6.82–6.77 (m, 3H, ArH); ^13^C NMR 188.24, 176.36, 172.62, 165.48, 161.86, 146.20, 144.80, 142.20, 132.94, 131.94, 130.96, 130.47, 129.66, 129.24, 129.12, 127.94, 127.45, 125.96, 125.10, 121.50, 120.98, 118.64, 116.58, 116.36; IR; 1620 (C=C); 1695 (C=O), 3065 (C=CH), 3420 (OH); Element analysis calculated of C_24_H_16_FN_3_O_2_, C, 72.54; H, 4.06; N, 10.57, present C, 72.60; H, 4.10; N, 10.60; EIMS: *m*/*z* (%): 397 (38.53) [M]^+^.

(E)-3-(4-(Dimethylamino)phenyl)-1-(3-((E)-(4-hydroxyphenyl)diazenyl)phenyl)prop-2-en-1-one (C13)

Dark brown-red crystals, mp (327–329 °C), ^1^H NMR at ẟ; 10.42 (s, IH, OH), 8.46–8.25 (m, 2H, ArH), 8.06–7.87 (m, 4H, ArH),7.78–7.70 (m, 4H, ArH),7 (d, *J* = 8.4 Hz, 2H, ArH), 6.77 (d, *J* = 7.6 Hz, 2H, ArH).3.03 (s, 6H, CH_3_); ^13^C NMR; 188.61, 161.79, 146.33, 140.02, 131.53, 130.28, 129.24, 128.36, 126.45, 125.86, 125.63, 122.30, 121.27, 119.58, 116.49, 116.33, 112.21, 39.35; IR; 1520 (N=N), 1580 (C=C), 1650 (C=O), 2870 (CH), 3070 (C=CH); Element analysis calculated of C_23_H_21_N_3_O_2_, C, 74.37; H, 5.70; N, 11.31: present C, 74.40; H, 5.70; N, 11.30; EIMS: *m*/*z* (%): 371 (50.56) [M]^+^.

(E)-1-(3-((E)-(2,4-Dihydroxyphenyl)diazenyl)phenyl)-3-(4-(dimethylamino) phenyl) prop-2-en-1-one (C14)

Dark brown-red crystals, mp (330–332 °C), ^1^H NMR; 13.37–13.25 (m, 2H, 2OH), 8.41 (s, 1H, ArH), 8.11 (d, *J* = 6.8 Hz, 1H, ArH), 8 (d, *J* = 7.6Hz, 1H, ArH), 7.77–7.56 (m,5H, ArH) 6.78–6.76 (m, 2H, ArH), 6.42–6.40(m, 2H, ArH), 6.12 (s, 1H, ArH), 3.11 (s, 6H, CH_3_), ^13^C NMR; 188.54, 162.78, 152.55, 146.18, 140.46, 134.56, 133.26, 131.49, 130.24, 128.78, 128.62, 126.12, 125.94, 125.42, 121.94, 118.98, 116.40, 116.24, 112.21, 39.34; IR (cm^−1^) 1520 (N=N), 1580 (C=C), 1690 (C=O), 2850 (CH), 3100 (C=CH); Element analysis calculated of C_23_H_21_N_3_O3, C, 71.30; H, 5.46; N, 10.85: present C, 71.30; H, 5.50; N, 10.90: EIMS: *m*/*z* (%): 387 (47.65) [M]^+.^.

5. -((E)-(4-((E)-3-(4-(Dimethylamino)phenyl)acryloyl)phenyl)diazenyl)-2-hydroxy benzoic acid (C15)

Dark red crystals, mp (decomposition 335 °C), ^1^H NMR; 14.25 (S, H, COOH), 11.24 (S, H, OH), 8.35 (s, 1H, ArH), 8.06 (d, *J* = 6.8 Hz, 1H, ArH), 7.78 (d, *J* = 7.6 Hz, 1H, ArH), 7.72–7.51(m,5H, ArH) 6.73–6.68 (m, 2H, ArH), 6.42–6.37 (m, 2H, ArH), 6.10 (s, 1H, ArH), 3.10 (s, 6H, CH_3_), ^13^C NMR; 187.42, 178.25, 161.68, 151.45, 145.16, 139.32, 133.44, 132.24, 130.29, 129.22, 128.42, 127.32, 126.52, 125.90, 125.38, 120.90, 117.78, 116.56, 116.14, 112.18, 39.32; IR (cm^−1^), 1520 (N=N), 1580 (C=C), 1690 (C=O), 2850 (CH), 3100 (C=CH); Element analysis calculated of C_24_H_21_N_3_O_4_, C, 69.39; H, 5.10; N, 10.11; Present C, 69.40; H, 5.10; N, 10.10: EIMS: *m*/*z* (%): 415 (39.73) [M]^+.^.

(E)-3-(4-(Dimethylamino)phenyl)-1-(3-((E)-(4-hydroxynaphthalen-1-yl)diazenyl) phenyl) prop-2-en-1-one (C16)

Pale brown crystals, mp (311–313 °C), ^1^H NMR 9.68 (s, 1H, OH), 8.70–8.14 (m, 4H, ArH), 8.09–7.44 (m, 5H, ArH), 7.40–6.79 (m, 7H, ArH), 3.06 (s, 6H, CH_3_); ^13^C NMR, 188.26, 162.45, 154.23, 144.28, 141.25, 140.29, 139.48, 136.48, 135.24, 134.25, 131.28, 130.26, 130.14, 129.26, 128.98, 128.54, 127.24, 126.94, 124.24, 121.23, 120.18, 118.24, 116.44, 39.42; IR (cm^−1^), 1520 (N=N), 1620 (C=C), 1695 (C=O), 2820 (CH), 3030 (C=CH); Element analysis calculated of C_27_H_23_N_3_O_2_, C, 76.94; H, 5.50; N, 9.97: present C, 76.90; H, 5.50; N, 9.90; O, 7.60; EIMS: *m*/*z* (%): 421 (28.86) [M]^+.^.

(E)-3-(4-(dimethylamino)phenyl)-1-(3-((Z)-(2-hydroxynaphthalen-1-yl)diazenyl) phenyl) prop-2-en-1-one (C17)

Dark red crystals, mp (322–324 °C), ^1^H NMR; at ẟ 15.73 (s, 1H, OH), 8.56–8.48 (m, 2H, ArH), 8.11–7.99 (m, 3H, ArH), 7.76–7.50 (m, 8H, ArH), 6.93–6.79 (m, 3H, ArH), 3.04 (s, 6H, CH_3_); ^13^C NMR 188.42, 170.72, 152.52, 146.35,145.77, 141.12, 140.30, 133.21, 131.73, 130.94, 129.94, 129.79, 129.52, 128.42, 127.74, 126.63, 124.74, 122.38, 122.06, 121.92, 119.40, 116.33, 112.21, 39.31; IR (cm^−1^), 1510 (N=N), 1570 (C=C), 1670 (C=O), 2840 (CH), 3020 (C=CH), 3420 (OH); Element analysis calculated of C_27_H_23_N_3_O_2_, C, 76.94; H, 5.50; N, 9.97; present C, 76.90; H, 5.50; N, 9.90: EIMS: *m*/*z* (%): 421 (19.44) [M]^+.^.

(E)-3-(4-(dimethylamino)phenyl)-1-(3-((Z)-(8-hydroxyquinolin-5-yl)diazenyl) phenyl) prop-2-en-1-one (C18)

Dark red crystals, mp (326–328 °C), ^1^H NMR at ẟ 9.38 (s, 1H, OH), 9.37 (d, *J* = 8 Hz, 1H), 9.02–8.62 (m, 2H, ArH), 8.30–8.23 (m, 3H, ArH), 8.09–8.07 (d, *J* = 8.8 Hz, 1H, ArH), 7.83–7.68 (m,6H, ArH), 7.29–7.27 (m, 1H, ArH), 6.79–6.77 (m, 1H, ArH), 3.04 (s, 6H, CH_6_). ^13^C NMR at ẟ 188.32, 171.24, 153.24, 145.95,145.64, 141.22, 140.38, 137.24, 131.96, 130.66, 129.42, 129.54, 129.42, 128.23, 127.56, 126.96, 125.14, 121.98, 121.34, 119.40, 116.41, 112.22, 39.33; IR (cm^−1^), 1505 (N=N), 1590 (C=C), 1685 (C=O), 3030 (C=CH), 3350 (OH); Element analysis calculated of C_26_H_22_N_4_O_2_, C, 73.92; H, 5.25; N, 13.26: present C, 73.90; H, 5.30; N, 13.30: EIMS: *m*/*z* (%): 422 (30.11) [M]^+.^.

3-Aminoacetophenone was diazotized with sodium nitrite in a strongly acidic environment using Conc. HCl. The prepared diazonium salt solutions were coupled with phenol (dissolved in KOH solution) in an ice bath to afford azo-derivatives A1–A6. The azo derivatives were prepared according to the reported methods. Compounds A1 and A5 were reported and prepared according to references [24,25].

There are two pathways for C7 to C18 synthesis. The first one, which included diazotization followed by condensation according to the report way, was used. The other synthetic pathway for C7 to C18 was conducted through Claisen–Schmidt followed by diazo coupling, but this pathway afforded a lower yield than the one used. Additionally, this pathway accompanied a percentage of byproducts that cannot be easily purified.

Moreover, the reported synthetic pathway for compounds A1 and A5 was applied to synthesize target compounds.

The novel azo derivatives’ structure elucidation was done using NMR, mass, and IR spectroscopy with element analysis.

The ^1^H NMR spectra of novel A2, A3, A4, and A6 showed characteristic aliphatic peaks at ẟ 2.51–2.52, equivalent to a methyl group, but the OH peaks at ẟ 8.74–15.60 matched the OH of either a phenol or acid.

In addition, the ^13^1NMR spectra of azo derivatives showed aliphatic (methyl group) and carbonyl peaked at ẟ 27.42–31.62 and 185.15–198.76, respectively. Azo-derivatives condensed in an alkaline medium with 4-fluorobenzaldehyde and 4-dimethylamino benzaldehyde to produce the new chalcones C7 to C12 and C13 to C18, respectively.

The ^13^C NMR peaks of C13 to C18 methyl groups appeared at ẟ 39.31–39.42, but the carbonyl group peaks of C7 to C18 appeared at ẟ 185.25–188.99.

Also, IR spectra of the novel compounds showed characteristic peaks of phenolic OH, carboxylic OH, and the carbonyl group of acetyl, carboxylic, and chalcone groups.

The newly synthesized compounds showed molecular ion peaks abundance ranging from 1.47 to 76.94.

### 2.2. Cytotoxic Activity

Cytotoxicity was conducted by applying the MTT assay method for all designed compounds on human malignant melanoma (A375), pancreatic ductal cancer (Panc-1), lung cancer (H-460), human colon cancer (HT-29), and pancreatic cancer (PaCa-2) (Table 1).

The IC_50_ was expressed in micromoles. Most of the tested compounds exhibited promising activity against applied cell lines, particularly compounds C9 and C10, which displayed potent effect, with IC_50_ values of 1.2 ± 0.3, 2.1 ± 0.3, 3.2 ± 1.1, 1.2 ± 0.2, and 1.8 ± 0.4 on HT-29, PaCa-2, A375, H-460, and Panc-1, respectively, for compound 9 and IC_50_ values of 1.1 ± 0.2, 0.9 ± 0.4, 2.6 ± 1.0, 1.2 ± 0.1, and 1.6 ± 0.5 µM against HT-29, PaCa-2, A375, H-460, and Panc-1, respectively, for compound 10 (Table 1). The presence of the 4-fluorophenyl moiety enhanced the cytotoxic effect of compounds C7–C12, and the presence of a -COOH group in compounds A3, C9, C15 was responsible for the promising impact of these compounds

### 2.3. Protein Kinase Inhibition Activity

**Figure 2:**
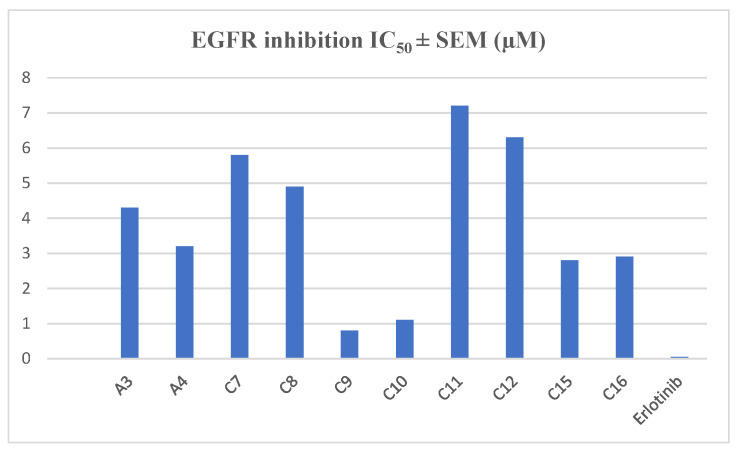
**SSAR9-B:** Effects of compounds on EGFR.

The cytotoxic mechanism of the designed compounds against tested cell lines was studied by evaluating their kinase inhibitory effect on EGFR. The results confirmed the potent impact of both C9 and C10 with IC_50_ values of 0.8 ± 0.3 and 1.1 ± 0.2 µM, respectively (Table 2, Figure 2).

The results showed that C9 had the most potent inhibitory activity among all tested compounds, which may be attributed to the presence of an α,β-unsaturated ketone, 4-fluorophenyl and carboxylic groups, which facilitated their binding with the enzyme through a hydrogen bond (HB), which was comparable to that of the standard Erlotinib drug (IC_50_ = 0.05 ± 0.02 µM) (Table 2, Figure 2).

### 2.4. Inhibition of Secretory PLA2-V, COX-1, COX-2 by Tested Compounds

**Figure 3:**
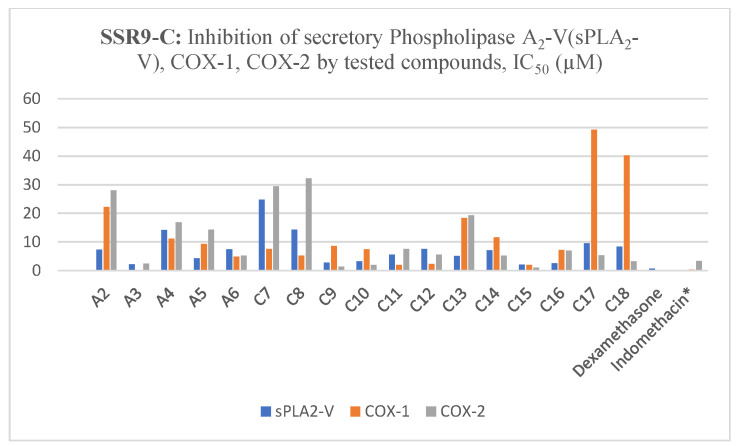
**SSR9-C:** Inhibition of secretory phospholipase A_2_-V (sPLA_2_-V), COX-1, COX-2 by tested compounds.

Assessment of the target compounds as sPLA2 inhibitors was conducted by Ellman’s method-based assay (Table 3), dexamethasone was applied as a positive control. The target compounds exhibited promising sPLA2 inhibitory effect ranging from IC_50_ 24.72 ± 1.59 (C7) to 2.01 ± 1.11 µM (C15). (Table 3, Figure 3)

The results revealed that compounds A3, C9, C10, C15, and C16 were the most potent derivatives. The presence of carboxyl groups in A3, C9, and C15 contributed to improving the activity against inflammatory enzymes but the presence of an α,β-unsaturated ketone and 4-fluorophenyl groups increased inhibitory activity with increasing selectivity towards COX-2 enzymes. In contrast to A3, compound A4 showed weak activity against inflammatory mediators, but upon condensation with 4-fluorobenzaldehye, compound C10 was produced and possessed a potent and selective effect against COX-2. This potent effect was attributed to the presence of an α,β-unsaturated ketone, 4-fluorophenyl groups, and the bulk volume produced from diazo-coupling with a-naphthol. The electron-withdrawing fluorine atom in C9 and C15 had a greater enhancing effect against the COX enzyme, compared to the electron-donating dimethylamino group in compounds C10 and C16. In both conditions, the α,β-unsaturated ketone moiety was found to enhance activity (Table 3, Figure 3).

### 2.5. Inhibition of Release of IL-6 and TNF-α in LPS-Stimulated Macrophages

**Figure 4:**
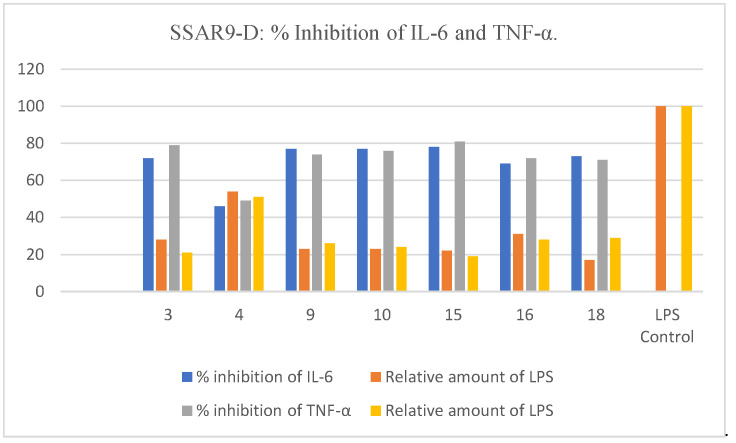
**SSAR9-D**: % Inhibition of IL-6 and TNF-α.

In the current study, inhibition of the release of liposaccharide (LPS)-triggered TNF-α and IL-6 in mouse macrophages (RAW264.7) was evaluated for the target compounds A3, A4, C9, C10, C15, C16, and C18, by applying the ELISA technique (Table 4, Figure 4). Most of the tested compounds exhibited promising activity with maximum inhibition rates of 49–81% and 46–78% against LPS-triggered expression of TNF-α and IL-6, respectively.

The highest inhibition effect of LPS-stimulated IL-6 and TNF-α (78 and 81%, respectively) was obtained by C15 as an LPS control. The presence of carboxylic groups in compounds A3, C9, and C15 potentiates an effect on IL-6 and TNF-α; this effect was increased in compounds C9 and C15 as a result of chalcone formation whether with 4-fluorophenyl or 4-dimethylaminophenyl (Table 4, Figure 4).

Tested compounds inhibited LPS-induced TNF-α and IL-6 secretion in RAW 264.7 macrophages. Cells were pretreated with synthetic compounds (10 µM) for 2 h, then treated with LPS (0.5 µg/mL) for 22 h.

### 2.6. Molecular Docking

The crystal structures of EGFR kinase (PDB ID: 1M17) and COX enzyme (PDB ID: 1CX2) were obtained from the Protein Data Bank (PDB) (https://www.rcsb.org/, accessed on 24 December 2021), prepared as a receptor by removing waters and co-crystallized ligands and ions then protonated using Pymol software (Ver. 2.5.1). Meanwhile, the 2D structure of the ligands was drawn through ChemDraw software and saved in mol format. The 2D structures were converted into 3D, then reduced, minimized, and optimized using MMFF94 force field through Avogadro software (Ver. 1.2.0) [26]. Blind docking was performed using a web-based program called CB-DOCK, accessed on 24 December 2021, and 15 and 16 January 2022 (http://clab.labshare.cn/cb-dock/php/). After submission, CB-Dock checked the input files and converted them to pdbqt-formatted files using OpenBabel and MGLTools. Next, CB-Dock predicted the cavities of the protein and calculated their centers and sizes of the top N (n = 5 by default). Each center and size, and the pdbqt files, were submitted to AutoDock Vina for docking. The final results were displayed after the computation of N rounds [27]. The benchmarks performed by Liu et al. (2020) illustrated success rates for top-ranking poses which had root mean squared deviation (RMSD) within 2 Å from the position in the X-ray crystal structure. CB-Dock outperformed other blind docking tools, and the profiles of interaction and visualization were performed for the best-docked complexes using Discovery Studio software (Ver. 21.1.0.20298) [28].

**Figure 5:**
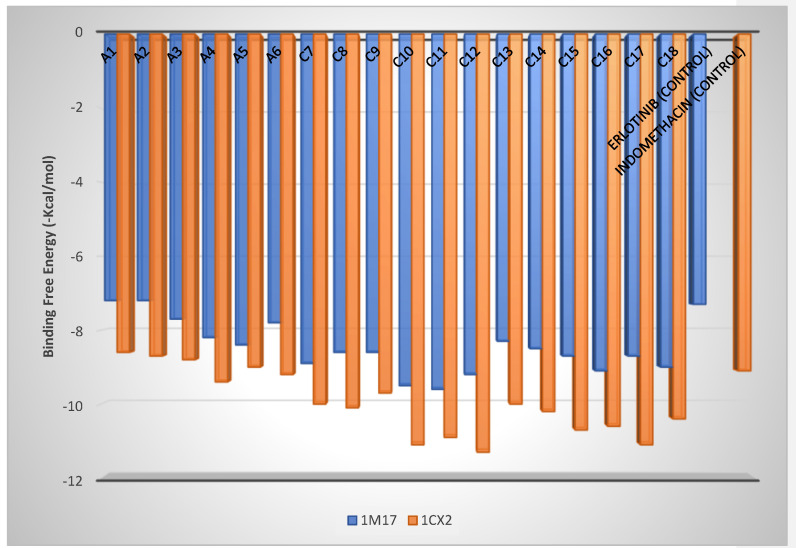
Binding free energy values were calculated by molecular docking of Azo derivatives (A1-A6) and Chalones (C7-C18) ligands with 1M17 and 1CX2 receptors.

The binding free energies (∆G) for the Azo derivatives (A1–A6) and Chalones (C7–C18) ligands docked at 1M17 and the 1CX2 receptors are shown in Figure 5, revealing the best poses obtained in the molecular-docking analyses. The lower the ∆G, the more significant the interaction between the receptor and the ligands with potential activity. Generally, Chalones C7–C18 displayed higher binding affinities than Azo derivatives (A1–A6). C9 at both receptors had high docking scores (−8.3 to −9.6): −9.6 kcal/mol at 1M17 and from −9.7 to −11.3 kcal/mol at 1CX2.

Among chalones, the 4-fluorophenyl moiety in C7–C12 seemed to be responsible for the higher binding free energies, which agreed with the cytotoxic and protein kinase inhibition on EGFR activities for C9 and C10. Again, all the chalone derivatives exhibited high docking scores with COX-2 (Figure 5).

High docking scores with COX-2 (Figure 5).

According to the above molecular docking results in Table 3, the potent derivatives for inhibiting COX-2 were C9, C10, C15, and C16. It is noteworthy that the similar backbone and presence of active groups, such as the carboxylic C15, seemed to be the main reasons for the comparable molecular docking results of C13–C18.

**Figure 6:**
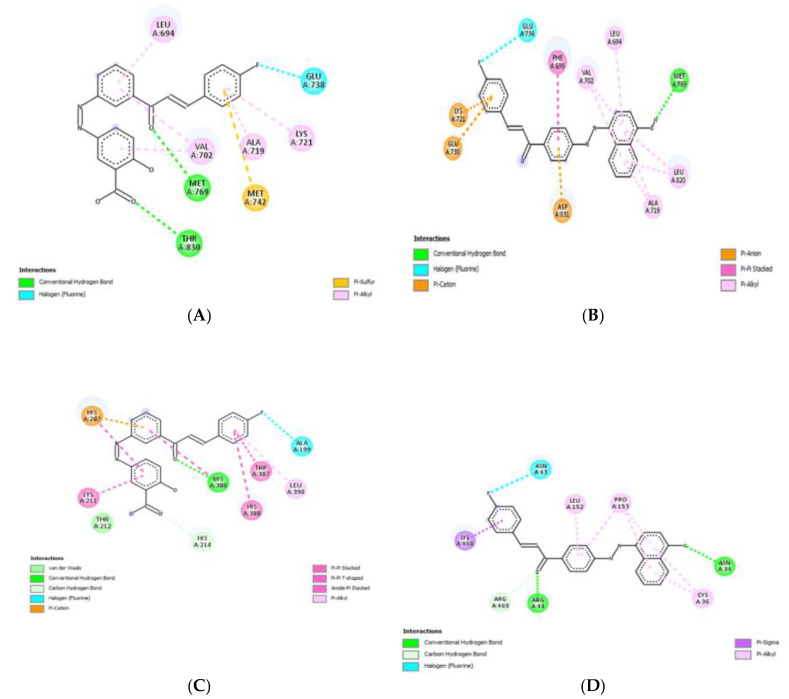
Interactions of ligands with 1M17 (**A**): C9, and (**B**): C10 and ligands with 1CX2 (**C**): C9 and (**D**): C10.

Figure 6a–d shows that the interaction of C9 and C10 ligands with 1M17 and 1CX2 receptors were the most potent in assays performed experimentally and had among the best docking scores. Compared with C9, the higher binding affinity of C10 with 1M17 was attributed to the conventional hydrogen bond formed with MET A:769, fluorine bonding with GLU A:734, pi–pi stacked with PHE A:699, and other unique bonds with many receptor residues: pi-cation, pi-anion, and pi-alkyl (Figure 6b). Conventional H-bonding with MET A:769 and THR A:830 residues of 1M17, in addition to halogen interaction at GLU A:738, were responsible for the comparable binding energy of C9. Still, the absence of other effective bonds was the main reason it had a lower affinity than C10. The number of conventional H-bonds with ARG A:44 and ASN A:34, and the unique pi-sigma interaction with LYS A:468, primarily involving charge transfer, helped firmly bind C10 to the 1CX2 receptor residues. Similar halogen (fluorine) interactions with ALA A:199 and ASN A:43 were shown for C9 and C10 in addition to the C–H bonds with HIS A:214, THR A:212, and ARG A:469 moieties (Figure 6c,d). Again, the types of interactions and number of specific bonds, such as conventional H-bonds, were the main reasons for the differences in binding affinity between C9 and C10 to the 1CX2 receptor.

## 3. Materials and Methods

### 3.1. Synthesis of Azo-Derivatives (A1–A6)

A mixture of 3-aminoacetophenone (1.35 g, 0.01 mol), conc. hydrochloric (5 mL), and solution of sodium nitrite (0.013 mol, 5 mL distilled water) were stirred in an ice bath for 6 h. The resulting cold diazonium clear solution was portion-wise added to an ice-cold alkaline solution of corresponding phenol derivatives (0.01 mol) in KOH (30%, 10 mL). The reaction mixture was stirred for 24 h. The resulting azo derivatives A1–A6 were neutralized using aqueous CH_3_COONa solution (10%). The precipitated azo derivatives A1–6 were filtered, collected, and filtered. The A1–A6 precipitates were purified using normal column silica gel chromatography by eluent solution CH_2_Cl_2_:CH_3_OH (in a ratio of 80:20 to 55:45) solvent system.

The synthesized A1–A6 color varied from dark red to brown, and the yield percentage ranged from 65 to 78%. Compounds 1 and 5 were prepared according to reported methods [24,25,29,30].

### 3.2. Synthesis of Chalcones C7–C18, General Method

To a mixture of ethanolic solution of azo-derivatives A1–A6 (0.01 mol) and 4-dimethylaminobenzaldehyde or 4-fluorobenzaldehyde (0.01 mol); NaOH (6 mL, 40%) was added dropwise at <10 °C. The reactants were stirred for 72 h at room temperature. Then the reaction solution was poured into ice-water, and the separated precipitate was filtered and crystallized from ethanol (70%).

The synthesized C7–C18 color varied from yellow to red, and the yield percentage ranged from 60 to 70%.

### 3.3. Cytotoxicity Assay

Cytotoxicity was conducted for all synthesized compounds (C1–C18) to determine their killing power of tumor cells, confirming their anticancer activity (Table 1). The standard cytotoxic Erlotinib was applied as control, and five different cell lines were applied and selected as stated in the Supplementary Materials [31,32,33].

### 3.4. EGFR Inhibitory Activity

To determine the cytotoxic mechanism of the active compounds, protein kinase inhibitory potential was assessed for the most functional products against the EGFR enzyme. As previously reported, the standard kinase screening protocol was used (Table 2). Further details are explained in the Supplementary Materials [34,35].

### 3.5. Assay of Secretory PLA2-V Activity

The standard procedure for Ellman’s photometric assay was applied for determining the secretory PLA2 activity [36] (See Supplementary Materials).

### 3.6. Cyclooxygenase Assay

COX-1 and COX-2 inhibitory activity was assessed for all target compounds by quantifying prostaglandin E2 (PGE2). All standard protocols were applied, and the experiment was conducted in triplicate (Table 3) [37,38] (See Supplementary Materials).

### 3.7. Cell Treatment and ELISA Assay for IL-6 and TNF-α

The reported method for testing IL-6 and TNF-α inhibitory activities was adopted (Table 4) [39] (See Supplementary Materials).

### 3.8. Statistical Analysis

All statistical analysis was performed by ANOVA software (One-way analysis of variance). P < 0.05 was approved as statistically significant.

### 3.9. Virtual Docking Study

COX-2 isoform and EGFR crystal structures were achieved from the PDB (COX-2 ID: 1CX2 and EGFR ID:1M17), and recognition of key amino acids required for enzyme inhibition ligands were recognized. The protein preparation of the enzyme and docking study of the tested compounds were conducted using Pymol software (Ver. 2.5.1) [8,27,32]. The experimental details will be presented in Supplementary Materials.

## 4. Conclusions

The main target of this research was the design of multi-target drugs oriented towards two critical enzymes COX-2 and EGFR. Compounds C9 and C10 showed potent activity against both enzymes, and this activity was attributed to the presence of an α,β-unsaturated ketone and 4-fluorophenyl or 4-dimethylaminophenyl. The carboxylic group and electron-withdrawing fluorine had a pronounced effect on C9 activity. A future plan for this work concerns in vivo studies for inflammation and cancer and also a study of the toxicity of synthesized compounds.

## Figures and Tables

**Table 1:** **SSAR9-A**: Effects of tested samples on cell lines.

N.	Code	Cell Viability %	Antiproliferative Activity IC_50_ ± SEM (µM)				
			HT-29	PaCa-2	A375	H-460	Panc-1
1.	A1	80.3 ± 1.6	8.3 ± 2.1	8.6 ± 1.2	9.3 ± 1.9	7.4 ± 2.2	9.1 ± 1.8
2.	A2	90.4 ± 1.9	9.2 ± 2.5	9.6 ± 1.5	8.4 ± 2.1	5.9 ± 2.4	9.6 ± 2.0
3.	A3	79.6 ± 4.5	1.8 ± 0.5	2.4 ± 1.5	4.0 ± 1.5	1.8 ± 0.4	2.2 ± 0.9
4.	A4	87.4 ± 1.7	2.7 ± 0.6	2.0 ± 0.6	4.4 ± 1.9	3.4 ± 1.0	2.0 ± 0.4
5.	A5	88.2 ± 1.2	11.9 ± 2.5	10.5 ± 1.9	9.6 ± 2.4	9.2 ± 1.2	9.6 ± 1.8
6.	A6	82.5 ± 1.7	7.6 ± 1.4	9.5 ± 1.9	9.5 ± 1.5	8.8 ± 2.3	6.9 ± 2.1
7.	C7	82.6 ± 1.2	4.5 ± 1.7	2.9 ± 1.7	5.3 ± 1.5	5.0 ± 1.4	4.8 ± 1.6
8.	C8	83.5 ± 1.2	2.5 ± 07	2.8 ± 0.6	2.6 ± 0.5	2.2 ± 0.5	1.9 ± 0.7
9.	C9	90.2 ± 4.1	1.2 ± 0.3	2.1 ± 0.3	3.2 ± 1.1	1.2 ± 0.2	1.8 ± 0.4
10.	C10	91.2 ± 2.9	1.1 ± 0.2	0.9 ± 0.4	2.6 ± 1.0	1.2 ± 0.1	1.6 ± 0.5
11.	C11	82.2 ± 2.4	2.9 ± 1.0	1.6 ± 0.5	3.5 ± 1.2	2.8 ± 0.7	1.6 ± 0.5
12.	C12	92.0 ± 2.0	1.4 ± 0.5	0.8 ± 0.4	2.4 ± 1.1	1.9 ± 0.3	1.2 ± 0.7
13.	C13	90.6 ± 1.7	7.8 ± 1.8	7.4 ± 1.7	8.4 ± 1.5	6.6 ± 1.2	8.7 ± 1.4
14.	C14	89.4 ± 2.1	6.5 ± 1.4	5.9 ± 1.9	4.9 ± 1.6	5.2 ± 1.3	4.6 ± 1.7
15.	C15	87.3 ± 2.1	1.6 ± 0.2	2.3 ± 0.4	3.4 ± 1.8	1.7 ± 0.2	1.9 ± 0.1
16.	C16	92.5 ± 2.0	1.8 ± 0.4	1.9 ± 0.5	2.8 ± 1.0	2.9 ± 1.0	1.8 ± 0.5
17.	C17	87.2 ± 1.4	9.2 ± 1.6	7.5 ± 1.9	8.8 ± 2.5	5.6 ± 2.1	2.5 ± 1.2
18.	C18	88.4 ± 2.9	6.7 ± 0.6	6.4 ± 1.5	8.8 ± 2.5	6.3 ± 1.4	6.4 ± 0.4
19.	Erlotinib	-	0.07 ± 0.04	0.06 ± 0.04	4.14 ± 1.2	0.04 ± 0.02	0.05 ± 0.02

**Table 2:** **SSAR9-B:** Effects of compounds on EGFR.

Compound	EGFR Inhibition IC_50_ ± SEM (µM)
A3	4.3 ± 0.7
A4	3.2 ± 0.8
C7	5.8 ± 1.1
C8	4.9 ± 0.9
C9	0.8 ± 0.3
C10	1.1 ± 0.0.2
C11	7.2 ± 1.4
C12	6.3 ± 1.7
C15	2.8 ± 0.5
C16	2.9 ± 0.4
Erlotinib	0.05 ± 0.02

**Table 3:** **SSR9-C:** Inhibition of secretory Phospholipase A_2_-V (sPLA_2_-V), COX-1, COX-2 by tested compounds.

Compound	sPLA_2_-V IC_50_ (µM)	COX-1 IC_50_ (µM)	COX-2 IC_50_ (µM)
A2	7.25 ± 1.24	22.2 ± 2.45	27.92 ± 1.65
A3	2.14 ± 0.94	0.21 ± 0.07	2.40 ± 0.8
A4	14.14 ± 1.52	11.12 ± 1.05	16.78 ± 2.43
A5	4.24 ± 1.16	9.23 ± 1.64	14.25 ± 1.79
A6	7.42 ± 1.41	4.85 ± 1.20	5.21 ± 1.2
C7	24.72 ± 1.59	7.52 ± 1.42	29.42 ± 1.73
C8	14.21 ± 1.32	5.14 ± 1.27	32.15 ± 1.40
C9	2.74 ± 1.24	8.57 ± 1.89	1.27 ± 0.3
C10	3.14 ± 0.64	7.37 ± 1.44	1.88 ± 0.4
C11	5.57 ± 1.19	1.95 ± 1.07	7.53 ± 0.9
C12	7.45 ± 1.23	2.24 ± 1.06	5.47 ± 0.8
C13	5.06 ± 1.20	18.29 ± 1.27	19.22 ± 1.52
C14	7.04 ± 1.29	11.55 ± 1.20	5.14 ± 1.1
C15	2.01 ± 1.11	1.9 ± 0.23	0.95 ± 0.2
C16	2.51 ± 1.05	7.10 ± 1.29	6.89 ± 0.8
C17	9.45 ± 1.67	49.11 ± 2.69	5.29 ± 1.1
C18	8.29 ± 0.88	40.17 ± 3.42	3.20 ± 0.6
Dexamethasone	0.57 ± 0.06	-	-
Indomethacin *	-	0.27 ± 0.04	3.29 ± 0.5

* 30 µM concentration, Values are the mean ± SD; n = 3.

**Table 4:** **SSAR9-D**: % Inhibition of IL-6 and TNF-α.

Compound	% Inhibition of IL-6	Relative Amount of LPS	% Inhibition of TNF-α	Relative Amount of LPS
3	72	28	79	21
4	46	54	49	51
9	77	23	74	26
10	77	23	76	24
15	78	22	81	19
16	69	31	72	28
18	73	17	71	29
LPS Control	0	100	0	100

## Supplementary Materials

The following supporting information can be downloaded online.

## Sample Availability

All compounds are available from the authors.
