# Supplementary material for: EGFR and COX-2 Dual Inhibitor: The Design, Synthesis, and Biological Evaluation of Novel Chalcones
Source: Molecules. 2022 Feb 9;27(4):1158. doi: 10.3390/molecules27041158 (PMC8876975; doi:10.3390/molecules27041158)
Supplement: Supplementary file 1 [file molecules-27-01158-s001.zip › molecules-1563200-supplementary.pdf]

Materials and Methods

1.1. Chemicals and reagents

All reagents and chemicals were purchased from Sigma-Aldrich, Merck and Acros Organics. Recombinant human PLA_2-V, 5,5-dithiobis (2-nitrobenzoic acid) (DTNB) and 1,2-bis(heptanoylthio)-phosphatidylcholine were supplied by Cayman Chemicals USA. HCl, KCl and $CaCl_2$ were purchased from Merck, Germany. A COX inhibitor screening kit purchased from Cayman Chemicals was used to study COX inhibitory activity. IL-6 and TNF-α levels were calculated using mouse IL-6 and TNF-α ELISA kits from Cayman Chemicals USA. Abcam (Cambridge, England) supplied mouse RAW 264.7 macrophages. Fetal bovine serum (FBS), lipopolysaccharide (LPS) and cell culture chemicals were purchased from Sigma Aldrich. DMSO was used to dissolve the compounds.

Melting points (mp) were determined on a Thomas-Hoover capillary apparatus and are uncorrected. Infrared (IR,) spectra were recorded. The nuclear magnetic resonance (NMR) determination was obtained with Varian Mercury (USA) 400 MHz spectrometer at 400 (^{1}H) and 100MHz (^{13}C) in a DMSO- d_6 solution (Mansoura University, Mansoura, Egypt). The spectra were recorded by the standard Bruker software. The EIMS spectra were measured by El/MS 502 mass spectrometer with a direct inlet system and operating at 70 eV.

1.2 Cytotoxicity assay

The cytotoxicity of the **18** target compounds and erlotinib was evaluated by MTT assay using HT-29, PaCa-2, A375, H-460 and Panc-1 cell lines. Cytotoxicity was also evaluated against the normal human fetal lung fibroblast cell line, MRC5. All cells were obtained from the ATCC. The five cancer cells were sub-cultured in RPMI-1640 media (10% FBS) while MRC5 was maintained in Eagle's minimum essential medium (EMEM, 10% FBS). All cell lines were maintained at 37 °C, 5% CO2, and 100% relative humidity and separately cultured in 96-well (3 × 103/well) for overnight incubation at 37 °C. Final compound concentrations were 0, 0.05, 0.5, 5, 25, and 50 µM in 0.1% DMSO (n = 3). Before the addition of MTT to each well, plates were incubated for 72 h. After the addition, plates were incubated for 3 h. The supernatant was then aspirated and DMSO was added to each well. Absorbance was measured with a multiplate reader (Biorad PR4100, USA). Optical density of the purple formazan A550 is proportional to the number of viable cells.

The concentration of a compound that results in 50% inhibition (IC_{50}) compared to control cell growth (100%) was determined. All experiments were repeated three times and GraphPad Prism was used as the software for analysis [1-3].

1.3 EGFR inhibitory activity

To determine whether any of the selected compounds can affect EGFR, profiling of compounds 3, 4, 7-12, 15 and 16 against EGFR target was performed by employing erlotinib as a positive reference drug. PBS Bioscience EGFR Kinase Assay Kit, catalog # 40321 was utilized in the assay and the standard protocol was followed. All samples and controls were tested in triplicates and the data enlisted and represent IC_{50} values compared to that of the control. IC_{50} data are the average of the three experiments ± SD [4, 5].

1.4 Secretory phospholipase A_2 -V ($sPLA_2$-V) activity assay

The enzyme human recombinant $sPLA_2$-V was supplier applied in the test. For assessment of $sPLA_2$ activity, derived from Ellman's method (photometric assay) was used. Free thiols were exposed through ester bond hydrolysis of 1,2-bis(heptanoylthio)-glycerophosphocholine by $sPLA_2$-V. The modification of DTNB to 2-nitro-5-thiobenzoic acid was triggered by these thiols. Aqueous buffer solution (pH 7.5) consisting of Triton-X 100 (280 BM), Tris (24 mM), $CaCl_2$ (9 mM) and KCl (94 mM) was used to continue the assay. Before the assay, $sPLA_2$-V and substrate were added in the assay buffer, whereas DTNB was added to Tris-HCl (pH 8) solution. The final concentration of DTNB and the enzyme was 87 BM and 100 ng/mL, correspondingly. The assay (containing respective test compound, DTNB and substrate solution) was performed at 25°C using 96-well microliter plates. The enzyme's 100% activity was quantified by only adding enzyme and substrate. The negative control used in the assay was DMSO (at a concentration of 1.7% v/v) [6].

1.5 Cyclooxygenase Assay

The activity of compounds on COX-1 and COX-2 was determined using a commercial kit which measured the level of prostaglandin E_2 (PGE_2). Sheep-derived COX-1 or human recombinant COX-2 and 1 μM heme were pre-incubated (at 37 °C) in 100mM Tris-HCl buffer (at pH 8.0) in water bath for 10 min. 10μl arachidonic acid (100μM final concentration in reaction) was added to start the reaction. 1M HCl was added to quench the reaction, followed by the measurement of PGE_2 level using ELISA. DMSO was used to dissolve the compounds and diluted to anticipated concentration with potassium phosphate buffer (100mM) at pH7.4, followed by transfer to a mouse

anti-rabbit IgG-coated 96-well microliter plate. The primary antibody (mouse anti PGE_2) and tracer prostaglandin acetylcholine esterase were added to the plates. Afterwards, the plates were kept at 25°C for 15 h, followed by the removal of reaction mixtures and washing with potassium phosphate buffer (10mM) comprising Tween 20 (0.05%). Subsequently, 200µl Ellman's reagent was added to all wells. The plate was incubated in dark for 60 min at 25°C till control wells produced an OD=0.3-0.8. A PGE_2 standard curve was plotted from similar plate which was used to calculate PGE_2 levels in compounds' presence. The data (%) were presented in comparison to control (solvent-treated sample). The assay was done in triplicate and the results usually agreed inside 10% [7].

1.6 Cell treatment and ELISA assay for TNF-α and IL-6

Mouse macrophages (RAW 264.7) were cultured in dulbecco's modified eagle's medium (augmented with 100 U/mL penicillin, 100 µg/mL streptomycin and 10% FBS) in a 5% CO_2 95% air atmosphere at 37°C. The macrophages were treated with vehicle control or compounds (10 µM) for 2 h, followed by LPS (0.5 µg/mL) treatment of 1 day. The cells and media were individually collected after treatment. The collected media were centrifuged for 10 min at 1000 rpm. Mouse IL-6 and TNF-α ELISA kits were used to determine IL-6 and TNF-α level in the media. The supernatant was collected after centrifugation and stored. The cells were first washed using PBS and then collected by lysis buffer (NaF 20 mM, SDS 0.1%, Na_3VO_4 200 mM, EDTA 2 mM, NaCl 150 mM, NP40 1%, Tris-HCl 20 mM). The mixture was vigorously shaken in lysis buffer at 0°C for 10 min. The total protein was collected after centrifugation (at 12,000 rpm for 30 min at 4°C), followed by the determination of concentrations. Tested compounds inhibited LPS-induced TNF-α and IL-6 secretion in RAW 264.7 macrophages. Cells were pretreated with synthetic compounds (10 µM) for 2 h, then treated with LPS (0.5 µg/ml) for 22 h [8].

References:

1. Gao, X.; Xu, Y.X.; Janakiraman, N.; Chapman, R.A.; Gautam, S.C. Immunomodulatory activity of resveratrol: Suppression of lymphocyte proliferation, development of cell-mediated cytotoxicity, and cytokine production. *Biochem. Pharmacol.* **2001**, *62*, 1299–1308.
2. Gao, X.; Kuo, J.; Jiang, H.; Deeb, D.; Liu, Y.; Divine, G.; Chapman, R.A.; Dulchavsky, S.A.; Gautam, S.C. Immunomodulatory activity of curcumin: Suppression of lymphocyte proliferation, development of cell-mediated cytotoxicity, and cytokine production in vitro. *Biochem. Pharmacol.* **2004**, *68*, 51–61.
3. Musa, A.; Elmaidomy, A.H.; Sayed, A.M.; Alzarea, S.I.; Al-Sanea, M.M.; Mostafa, E.M.; Hendawy, O.M.; Abdelgawad, M.A.; Youssif, K.A.; Refaat, H. Cytotoxic Potential, Metabolic Profiling, and Liposomes of Coscinoderma sp. Crude Extract Supported by in silico Analysis. Int. J. Nanomed. 2021, 16, 3861.
4. Al-Sanea, M.M. Synthesis and biological evaluation of small molecule modulators of cdk8/cyclin c complex with phenylaminoquinoline scaffold. PeerJ. 2020, 8, e8649.
5. Al-Sanea, M.; Abdelazem, A.; Park, B.; Yoo, K.; Sim, T.; Kwon, Y.; Lee, S. ROS1 kinase inhibitors for molecular-targeted therapies. Curr. Med. Chem. 2016, 23, 142–160.
6. Pohanka, M.; Hrabinova, M.; Kuca, K. Diagnosis of intoxication by the organophosphate vx: Comparison between an electrochemical sensor and ellman s photometric method. Sensors 2008, 8, 5229–5237.
7. Ahmad, W.; Kumolosasi, E.; Jantan, I.; Bukhari, S.N.; Jasamai, M. Effects of novel diarylpentanoid analogues of curcumin on secretory phospholipase A2, cyclooxygenases, lipo-oxygenase, and microsomal prostaglandin E synthase-1. *Chem. Biol. Drug Design* **2014**, *83*, 670–681.
8. Gogos, C.A.; Drosou, E.; Bassaris, H.P.; Skoutelis, A. Pro-versus anti-inflammatory cytokine profile in patients with severe sepsis: A marker for prognosis and future therapeutic options. *J. Infect. Dis.* **2000**, *181*, 176–180.
